# Taxane-based Chemotherapy Induced Androgen Receptor Splice Variant 7 in Patients with Castration-Resistant Prostate Cancer: A Tissue-based Analysis

**DOI:** 10.1038/s41598-019-53280-5

**Published:** 2019-11-14

**Authors:** Myungsun Shim, Yunlim Kim, Yangsoon Park, Hanjong Ahn

**Affiliations:** 1 Department of Urology, Hallym University College of Medicine, Hallym University Sacred Heart Hospital, 22, Gwanpyeong-ro 170beon-gil, Dongan-gu, Anyang-si, Gyeonggi-do 14068 South Korea; 2 Institute for Innovative Cancer Research, Asan Institute for Life Science, Asan Medical Center, 88, Olympic-ro 43-gil, Songpa-gu, Seoul 05505 South Korea; 3 Department of Pathology, University of Ulsan College of Medicine, Asan Medical Center, 88, Olympic-ro 43-gil, Songpa-gu, Seoul 05505 South Korea; 4 Department of Urology, University of Ulsan College of Medicine, Asan Medical Center, 88, Olympic-ro 43-gil, Songpa-gu, Seoul 05505 South Korea

**Keywords:** Prognostic markers, Prostate

## Abstract

In total, 95 prostate cancer (Pca) patients who underwent transurethral resection of the prostate from 2000 to 2013 were assigned to four groups: Group 1, hormone-naïve and T1a or T1b Pca (n = 17); Group 2, hormone-sensitive and metastatic Pca (n = 33); Group 3, chemo-naïve castration-resistant Pca (CRPC), (n = 18); and Group 4, CRPC with chemotherapy (n = 27). Full-length androgen receptor (ARfl) transcript levels significantly increased from Group 1 through to Group 3 (*p* = 0.045), but decreased from Group 3 through to Group 4. AR splice variant 7 (ARV7) and glucocorticoid receptor (GR) transcript levels significantly increased from Group 1 through to Group 4 (*p* = 0.002 and 0.049, respectively). Kaplan–Meier curve revealed that the high transcript level of these three receptors resulted in significantly poorer cancer-specific survival (CSS) than that by low transcript level, although Cox regression analysis revealed that the ARV7 level alone was an independent prognostic factor for CSS in CRPC patients (high vs. low: hazard ratio, 1.897; 95% confidence interval, 1.102–3.625; *p* = 0.042). In conclusion, ARV7 and GR transcript levels significantly increase as Pca progresses to CRPC.

## Introduction

Androgen deprivation therapy (ADT) has been widely considered to be the primary treatment for metastatic prostate cancers (Pca). Although patients are initially responsive to ADT, the remission persists for only 2–3 years^1^; subsequently, the tumor progresses to castration-resistant Pca (CRPC), which is refractory to ADT. Several reports have described the process of castration-resistance acquisition, but its exact mechanism remains unclear.

Androgen signalling is known to remain active after progressing to CRPC^2^, and the androgen receptor (AR) in CRPC remains active during ADT^3,4^.Among the various mechanisms, a truncated spliced variant of AR that lacks the ligand-binding C-terminal but retains the transactivating N-terminal domain due to alternative splicing or non-sense mutations of the AR gene^5–8^, is believed to be one of the most important mechanisms responsible for generating constitutively active receptors that can activate target genes without ligand-binding^8,9^.

In addition to AR splice variant formation, previous studies have suggested that abnormal hyperactivation of glucocorticoid(GR) signalling contributes to the progression of hormone-dependent Pca to CRPC in patients receiving ADT^10,11^. Although glucocorticoids are known to produce some beneficial effects by acting as a pituitary suppressant that reduces adrenal androgen production^1^,the GR signalling pathway may play a different role in CRPC by promoting cancer progression through enhancement of the expression of certain pro-inflammatory cytokines, such as interleukin-6^10,12^.

Therefore, here we aimed to assess the expression of full-length AR (ARfl) and its most studied variant, termed AR splice variant 7 (ARV7), along with the GR during various Pca stages, based on the exposure and/or sensitivity to ADT and chemotherapy, using tissues obtained from human surgical specimens. We also evaluated the association between the transcript levels of each receptor and the durations of ADT as well as clinical outcomes.

## Results

### Patient characteristics

The median age, BMI, and serum PSA levels of the patients at surgery were 73.0 years, 23.7 kg/m^2^, and 46.0 ng/ml respectively. As expected, the patients tended to have tumors with a high GS and advanced clinical stage, which increased from Group 1 through to Group 4 (all *p* < 0.001). The clinical and pathological characteristics are summarized in Table 1.

**Table 1:** Comparison of the preoperative variables among the groups.

Variables	Group 1 n = 17	Group 2 n = 33	Group 3 n = 18	Group 4 n = 27	*p*
Age (years)	76.2 ± 7.6	71.8 ± 8.8	72.9 ± 9.4	71.1 ± 8.4	0.093
Body mass index (kg/m^2^)	24.4 ± 3.5	23.3 ± 3.6	24.4 ± 2.6	22.9 ± 3.0	0.305
Serum PSA level (ng/mL)^a^	37.3 ± 41.6	100.9 ± 114.5	40.0 ± 39.4	159.5 ± 218.2	0.024
Serum testosterone level (ng/mL)^a^	3.6 ± 1.6	0.3 ± 0.3	0.2 ± 0.2	0.3 ± 0.5	<0.001
Gleason score (%)					<0.001
7	7 (41.2)	4 (12.1)	0 (0.0)	0 (0.0)	
8	4 (23.5)	4 (12.1)	2 (11.1)	2 (7.4)	
9	5 (29.4)	19 (57.6)	7 (38.9)	15 (55.6)	
10	1 (5.9)	6 (18.2)	9 (50.0)	10 (37.0)	
Distant metastasis (%)	0 (0.0)	32 (97.0)	17 (94.4)	26 (96.3)	<0.001
ADT duration before surgery (months)^b^	—	2.8 ± 6.8	32.9 ± 31.3	35.7 ± 33.2	<0.001

Group 1, hormone-naïve and T1a or T1b prostate cancer (Pca); Group 2, hormone-sensitive and advanced/metastatic Pca; Group 3, chemo-naïve castration-resistant Pca (CRPC); Group 4, CRPC with chemotherapy.Values are presented as mean ± standard deviation or number (%).

PSA, prostate-specific antigen; ADT, androgen deprivation therapy.

^a^Measured within 1 week of surgery

^b^Excluded patients without androgen deprivation therapy

### Transcript levels of each receptor at different stages of Pca

**Figure 1:**
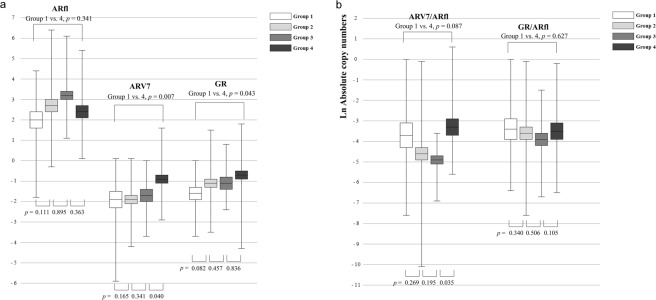
Mean values of absolute copy numbers of mRNA transcripts for (**a**) full-length androgen receptor (ARfl), androgen receptor splice variant 7 (ARV7), and glucocorticoid receptor (GR), (**b**) ARV7/ARfl and GR/ARfl ratios in each group. The copy numbers of the mRNAs of each receptor were adjusted to the corresponding housekeeping gene (GAPDH) mRNA levels and were transformed by the natural logarithm. Group 1, hormone-naïve and T1a or T1b prostate cancer (Pca); Group 2, hormone-sensitive and advanced/metastatic Pca; Group 3, chemo-naïve castration-resistant Pca (CRPC); Group 4, CRPC with chemotherapy.

The mRNA transcripts of ARfl, ARV7, and GR were detected, and their levels were quantified as copy numbers (transformed by the natural logarithm) using quantitive RT-PCR analysis. The mean transcript levels of all three receptors were lowest in tissues from Group 1, whereas those of ARV7 and GR specifically exhibited a significant increase from Group 1 through to Group 4 (*p* = 0.002 and 0.049, respectively, Fig. 1a), thus indicating that Group 4 patients demonstrated the highest transcript level. However, ARfl transcript levels increased from Group 1 through to Group 3 (*p* = 0.045), and decreased from Group 3 through to Group 4, although this decrease was not statistically significant (*p* = 0.363, Fig. 1a). Consistently, correlation between ARfl and ARV7 was significant Group 1, 2, and 3 patients (r = 0.273, *p* = 0.031) while it was not in group 4 patients (r = 0.175, *p* = 0.285, data not shown). In addition, the ARV7/ARfl ratio exhibited a large increase from Group 3 through to Group 4 (*p* = 0.035), although no significant change was observed from Group 1 through to Group 3 (*p* = 0.174, Fig. 1b). The GR/ARfl ratio was not significantly different among the groups (*p* = 0.266). The transcript level of each receptor and the serum testosterone level showed no correlation in patients receiving ADT (Groups 2, 3, and 4, data not shown).

### The ADT duration and the transcript level of each receptor

We also determined ADT duration in each patient and examined the transcript levels of all three receptors as well as their ratios to evaluate the effect of ADT duration on receptor transcription levels. ADT duration showed no correlation with ARfl (r = –0.056, *p* = 0.685) and GR (r = 0.102, *p* = 0.460) transcript levels. However, as ADT continued, the ARV7 transcript level tended to increase, even though the correlation coefficient was relatively small (r = 0.283, *p* = 0.036; Table 2). Furthermore, ADT duration demonstrated a stronger positive correlation with the ARV7/ARfl ratio than with ARV7 alone (r = 0.316, *p* = 0.019; Table 2), and no correlation with the GR/ARfl ratio (r = 0.118, *p* = 0.390).

**Table 2:** Correlation between the copy numbers of the mRNAs of each receptor and the duration of androgen-deprivation therapy.

	ARfl	ARV7	GR	ARV7/ARfl	GR/ARfl
**ADT duration**					
Coefficient	− 0.056	**0**.**283**	0.102	**0**.**316**	0.118
*p* value	0.685	**0**.**036**	0.460	**0**.**019**	0.390

Statistically significant values are indicated in bold.

ADT, Androgen-deprivation therapy; ARfl, full length androgen receptor; ARV7, androgen receptor splice variant 7; GR, glucocorticoid receptor.

### The effect of chemotherapy on the expression of each receptor in CRPC cell line

**Figure 2:**
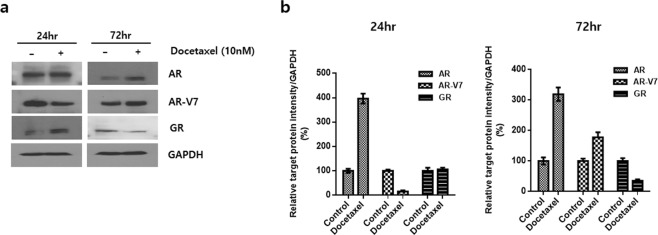
Changes of protein expression of full-length androgen receptor (ARfl), androgen receptor splice variant 7(ARV7), and glucocorticoid receptor (GR) after 24 and 72 hours of docetaxel treatment. 22Rv1 cells were treated with 10 nM of docetaxel for 24 and 72 hours. (**a**) Western blot analysis with ARfl, ARV7 and GR antibodies, GAPDH was used for loading control. (**b**) Relative protein expression level was quantified using Image-J software and normalized to GAPDH (n = 3).

To identify the effect of chemotherapy on the receptor status in CRPC, protein expression levels for each receptor in 22Rv1 cells 24 and 72 hours after docetaxel treatment were measured. We confirmed different changing patterns induced by chemotherapy according to each receptor that treatment with docetaxel increased the expression levels of ARfl, and ARV7 while reduced those of GR (Fig. 2). Notably, 24-hour treatment with docetaxel paradoxically decreased ARV7 expression and subsequent treatment for 74 hours increased the expression.

### CSS according to the transcript level of each receptor

**Figure 3:**
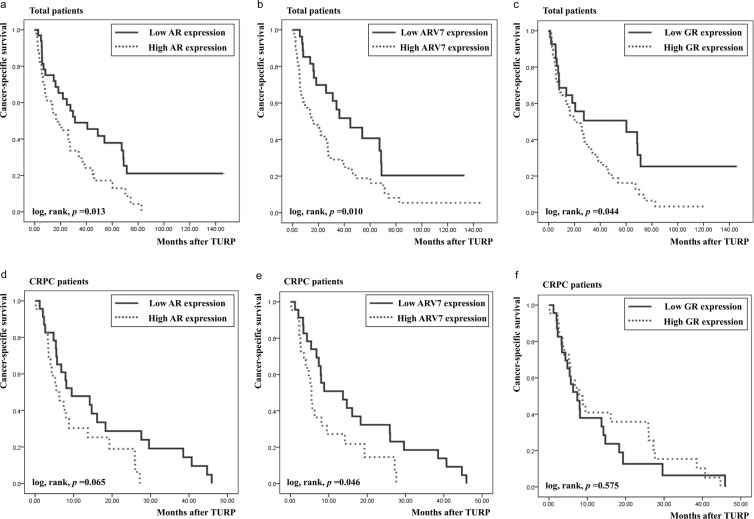
Cancer-specific survival according to the expression status of each receptor, as estimated using the Kaplan–Meier method. (**a**) Total, full-length androgen receptor (ARfl), (**b**) Total, androgen receptor splice variant 7(ARV7), (**c**) Total, glucocorticoid receptor (GR), (**d)** castration-resistant prostate cancer (CRPC), ARfl, (**e**) CRPC, ARV7, (**f**) CRPC, GR.

Kaplan–Meier curves indicated that a significantly poorer 5-year CSS was associated with high transcript levels of all three receptors than their low transcript levels (ARfl, 17.2% vs. 37.9%, *p* = 0.013, Fig. 3a; ARV7, 18.8% vs. 40.7%, *p* = 0.010, Fig. 3b; GR, 16.2% vs. 50.5%, *p* = 0.044, Fig. 3c). However, a sub-analysis of the CRPC patients indicated that only ARV7 transcript levels were associated with a significant survival difference (*p* = 0.046, Fig. 3e), whereas ARfl and GR transcript levels exhibited no survival difference (*p* = 0.065, Fig. 3d and *p* = 0.575, Fig. 3f, respectively). The multivariate analysis of all patients with Pca showed that the serum PSA level, GS of tumors, and distant metastasis were independent predictors of CSS (*p* = 0.046, 0.011, and 0.001, respectively, Table 3), whereas mRNA levels of all three receptors were not. However, in the sub-analysis of the CRPC patients, the ARV7 transcript levels remained an independent predictive factor (hazard ratio, 1.897; 95% confidence interval, 1.102–3.625; *p* = 0.042) along with the serum PSA levels (*p* = 0.028). The multivariate analysis revealed that GS 10 exhibited significantly poorer survival than GS 8 (*p* = 0.038) but not GS 9 (*p* = 0.097; Table 3).

**Table 3:** Univariate and multivariate Cox regression analysis of the factors predicting cancer-specific survival.

Variables	Univariate	Multivariate		
	HR (95% CI)	*p*	HR (95% CI)	*p*
**Total patients**				
Age (years)	0.982 (0.955–1.008)	0.175		
PSA level at surgery (ng/mL)	**1**.**002 (1**.**001–1**.**004)**	**0**.**007**	**1**.**002 (1**.**000–1**.**003)**	**0**.**046**
Testosterone level at surgery (ng/mL)	0.520 (0.307–0.882)	0.015	0.666 (0.397–1.119)	0.125
Gleason score		<**0**.**001**		**0**.**011**
8 vs. 7	3.346 (0.722–15.510)	0.123	**6**.**384 (1**.**168–36**.**953)**	**0**.**032**
9 vs. 7	**8**.**827 (2**.**098–37**.**133)**	**0**.**003**	**7**.**358 (1**.**569–41**.**238)**	**0**.**019**
10 vs. 7	**14**.**555 (3**.**308–64**.**035)**	<**0**.**001**	**13**.**231 (2**.**652–72**.**194)**	**0**.**002**
Distant metastasis	**11**.**247 (4**.**000–31**.**623)**	<**0**.**001**	**5**.**291 (2**.**235–23**.**822)**	**0**.**001**
ARfl (High vs. Low)	1.923 (1.141–3.240)	0.014	1.638 (0.895–3.159)	0.102
ARV7 (High vs. Low)	2.041 (1.174–3.548)	0.011	1.238 (0.953–4.328)	0.078
GR (High vs. Low)	1.787 (1.009–3.165)	0.047	0.953 (0.398–1.890)	0.725
**Patients with castration-resistant prostate cancer**				
Age (years)	1.001 (0.966–1.037)	0.962		
PSA level at surgery (ng/mL)	1.002 (1.001–1.004)	0.008	1.002 (1.000–1.005)	0.028
Testosterone level at surgery (ng/mL)	2.483 (0.837–7.361)	0.101		
Gleason score		0.126		0.106
9 vs. 8	3.973 (0.895–17.640)	0.070	3.847 (0.798–16.824)	0.097
10 vs. 8	4.933 (1.059–22.973)	0.042	4.982 (1.107–24.128)	0.038
Distant metastasis	3.923 (0.883–17.422)	0.072		
ARfl (High vs. Low)	1.852 (0.951–3.606)	0.070		
ARV7 (High vs. Low)	2.032 (1.163–3.782)	0.020	1.897 (1.102–3.625)	0.042
GR (High vs. Low)	0.838 (0.450–1.559)	0.576		

Statistically significant values are indicated in bold.

HR, hazard ratio; CI, confidence interval; ARfl, androgen receptor full length; ARV7, androgen receptor splice variant 7; GR, glucocorticoid receptor.

## Discussion

In the present study, we found that ARV7 transcript levels in human Pca tissue increased as the tumor progressed from hormone-naïve Pca to advanced Pca and finally to CRPC. Moreover, although ARV7 transcript levels increased at every stage from ADT administration (from Group 1 through to 2), to tumors becoming resistant to ADT (from Group 2 through to 3) and to chemotherapy administration (from Group 3 through to 4), the increase during the last stage was the greatest (*p* = 0.040, Fig. 1a). In addition, the ARV7/ARfl ratio exhibited a marked increase from Group 3 through to 4 which was the greatest (*p* = 0.035, Fig. 1b), thus indicating that ARV7 may play an important role in the development of resistance to chemotherapy. This can be further supported by our findings that showed docetaxel treatment increased ARV7 expression while reduced GR expression in CRPC cell lines (Fig. 2). Our result also can give explanations to earlier findings that the clinical activity of enzalutamide can be blunted by previous docetaxel chemotherapy^13–15^. Although initiation of taxane-based chemotherapy may not promptly induce ARV7, long-term exposure to chemotherapy may otherwise induce ARV7, which results in acquisition of resistance to subsequent AR-targeted therapy. Our results from CRPC cell lines showing that longer duration of treatment (72 hr) markedly increased the expression of ARV7, whereas short duration (24Hr) paradoxically decreased ARV7 expression (Fig. 2) may further support a delayed induction of ARV7 during chemotherapy. This can be further supported by our result Moreover, ARV7 transcript levels increased with an increase in ADT duration (Table 2), suggesting that formation of AR splice variant is facilitated by ADT initiation. On the other hand, transcript level of ARfl and ARV7 was not correlated in group 4 patients, suggesting that mechanisms driving ARfl increase may differ from those required for ARV7 generation which is consistent with the previous literatures^16–18^. Accordingly, increasing trend of ARfl from Group 1 through to 3 changed from Group 3 though to 4, also suggesting that ARfl played an important role in CRPC development while its role for gaining resistance to taxane-based chemotherapy is limited.

Another possible explanation for castration-resistance acquisition is the presence of tumor cells that can activate down-stream transcription without the presence of the AR, such as GR signalling as an alternate pathway^10,12,19^. Several previous reports have discussed the controversial role of the GR, and no consensus has been reached on whether the GR contributes to CRPC progression^20–22^. Our previous report has shown that the GR is strongly detected in DU145 cells, but not in LNCaP cells, and that dihydrotestosterone (DHT) activates the signal transducer and activator of transcription 5 (STAT5), thus enhancing cell proliferation, regardless of the presence of the AR^23^. These findings suggested that DHT uses the GR to activate STAT5 and bypass the AR signalling pathway. Based on our findings from examining actual human Pca tissues *in vivo*, GR transcript levels increased as the tumor progressed from hormone-naïve Pca to CRPC, suggesting that the GR plays a role in CRPC development. Hence, we postulated that CRPC cells gains continuous viability partly by activating the GR pathway as an alternative to the AR signalling pathway, in an androgen-deprived environment. However, once CRPC develops, the role of GR may be limited and ARV7 may then play an important role in ensuring tumor survival, progression, and gaining resistance to chemotherapy.

In our study, CRPC patients were further divided into two groups, namely chemo-naïve CRPC (Group 3) and CRPC with chemotherapy (Group 4), to investigate the change in receptor transcript levels due to docetaxel administration. We have also examined the changes of expression status of each receptor at protein level before and after docetaxel treatment in CRPC cell lines, and therefore, to investigate not only castration-resistance acquisition but also to exam the mechanism of resistance to chemotherapy in CRPC. The process of castration- and chemo-resistance acquisition in Pca may be summed up as follows, based on our current findings. First, when ADT is administered in hormone-naïve Pca (from Group 1 through to Group 2), Pca cells may not only increase ARfl levels along with ARV7 levels but also utilize the GR as an alternative pathway to adapt to the androgen-depleted environment, as transcript levels of all three receptors increased from Group 1 through to Group 2 (Fig. 1a). Second, as ADT continues, Pca becomes CRPC by a sustained increase in or maintenance of high levels of ARfl, ARV7 and GR transcription (from Group 2 through to Group 3, Fig. 1a). Finally, when chemotherapy is administered to treat CRPC, Pca cells may dramatically increase ARV7 transcript levels rather than ARfl transcript levels, which is not believed to be required in the process of chemo-resistance acquisition (from Group 3 through to Group 4, Fig. 1a). The importance of ARV7 in chemo-resistance is supported by our finding that ARV7/ARfl ratio was greatly increased from Group 3 through to Group 4 (*p* = 0.035, Fig. 1b) and docetaxel treatment increased the expression of ARV7 (Fig. 2). These findings are consistent with the results from previous literatures that ARV7 expression is associated with taxane resistance^16,24^. On the contrary, it seems that the role of the GR in the process of gaining chemo-resistance is limited, as the GR/ARfl ratio did not increase significantly from Group 3 through to Group 4 (Fig. 1b).

We demonstrated that among the receptors that we evaluated, transcript levels of only ARV7 remained independently predictive of a poor CSS in CRPC patients. The poor prognosis of patients with high ARV7 transcript levels is probably associated with the postulated constitutive activity of this variant. The fact that ARV7 was an independent prognostic factor only in CRPC patients and not in total Pca patients implies that high ARV7 transcript levels are associated with poor survival in CRPC patients and that ARV7 expression significantly enhances cell survival and cancer progression of CRPC. Hence, estimating the ARV7 expression level may provide useful information for predicting the prognosis of CRPC patients.

To the best of our knowledge, this is the first study to simultaneously describe the transcription pattern of ARfl, ARV7, and GR, and to demonstrate the expression of these receptors in individual patients at various Pca stages, including hormone-naïve, hormone-sensitive, metastatic Pca, and CRPC. Another advantage of our present study is that these expression patterns were directly examined using clinical Pca tissues derived from TURP specimens rather than *in vitro* cell lines.

There are some limitations in our study which includes the retrospective nature of the analysis, intratumoral heterogeneity is not considered although the possibility is likely exist^25^, protein expression levels from each patient were not examined because the amount of tissues of some patients were not enough to extract protein for the appropriate analyses. However, results of protein expression analyzed by immunohistochemistry and Western blot from a few patients are included in the supplementary.

In conclusions, our current findings suggested that in the absence of androgens, Pca cells convert ARfl into the constitutively active, ligand-binding domain truncated splice variant 7, and uses the GR pathway as an alternative pathway to maintain their viability. In addition, ARV7 may play an important role in acquiring resistance to chemotherapy in CRPC patients. Moreover, the ARV7 level may have some prognostic value because it was an independent predictor of CSS in CRPC patients.

## Materials and Methods

### Patients

The Institutional Review Board of the Asan Medical Center approved and supervised this study. All research was performed in accordance with relevant guidelines/regulations, and informed consent was obtained from all participants and/or their legal guardians. The medical records and pathological data of 124 Pca patients who underwent transurethral resection of the prostate (TURP) at our institution during 2000–2013 were reviewed. These patients included those who underwent TURP because of lower urinary tract symptoms and were subsequently diagnosed with Pca (clinical stage T1a or T1b) and those with advanced/metastatic Pca who underwent palliative surgery for bladder outlet obstructions. After excluding the patients (detailed exclusion criteria are shown in the supplementary information), 95 patients were finally included in the analysis. These patients were assigned to four groups: Group 1, hormone-naïve and T1a or T1b Pca (n = 17); Group 2, hormone-sensitive and advanced/metastatic Pca (n = 33); Group 3, chemo-naive CRPC (n = 18); and Group 4, CRPC with docetaxel chemotherapy (n = 27). The mean duration of ADT and chemotherapy was 30.1 ± 15.9 and 11.4 ± 8.4 weeks, respectively. The main aim of this study was to investigate the process by which castration resistance is acquired, by categorizing the Pca patients into groups from hormone-naïve Pca to CRPC and evaluating the difference in ARfl, ARV7, and GR transcript levels in these groups.

### Outcomes

Patient age, body mass index (BMI), serum prostate-specific antigen (PSA) and serum testosterone levels at surgery, Gleason scores (GS), distant metastasis, and ADT duration before surgery were assessed. Serum PSA and testosterone levels were evaluated within 1 week after surgery. ADT duration was estimated by subtracting the discontinued ADT duration from the total ADT duration before surgery if the patients received intermittent ADT. The primary objective of this part of the study was to evaluate ARfl, ARV7, and GR transcript levels in the groups. The secondary objective was to analyse the association between these levels and ADT duration as well as oncological outcomes.

### Quantitative reverse transcript polymerase chain reaction assay

After histological sectioning at multiple levels, the TURP specimens were routinely fixed and examined microscopically. RNA was extracted using the RNeasy FFPE kit (Qiagen, Inc., Tübingen, Germany). A quantitative reverse transcript polymerase chain reaction (RT-PCR) assay was used to evaluate the transcript level of each receptor. Receptors transcript levels are shown as the copy number of the messenger RNA (mRNA), adjusted to the corresponding housekeeping gene (GAPDH) mRNA levels. The 2*2^(−ΔΔCt)^ method was used to estimate the AR copy number, as previously described^26^. Details of mRNA extractions and RT-PCR are provided in the supplementary information.

### Western blot analysis

To observe the effect of chemotherapy on receptors at protein level in CRPC cells in *vitro*, we treated docetaxel for 24 and 72 hours to 22Rv1 cells and expression levels of receptors were identified suing Western blot. Detailed methods for cell culture and Western blot analysis are also provided in the supplementary information.

### Statistical analysis

Statistical comparisons between the groups were performed using the Student’s *t-*test for continuous variables and the chi-squared test (χ^2^) for categorical variables. The relationship between ADT duration and the transcript level of each receptor was assessed using the Spearman correlation coefficient. All potential factors, including receptor transcript levels associated with cancer-specific survivals (CSSs), were analysed using Kaplan–Meier analysis (univariate) and the Cox proportional hazards model (multivariate). Details of “high transcription” and “low transcription” are presented in the supplementary information. All tests were conducted using SPSS^®^ for Windows, version 15.0 (Statistical Package for Social Sciences ^TM^, Chicago, IL), and a *p*-value of < 0.05 was considered to be statistically significant.

## Supplementary information

Supplementary

## Data Availability

Raw data were generated at Asan Medical Center. Derived data supporting the findings of this study are available from the corresponding author [H.A.] on request.
