# Supplementary material for: Taxane-based Chemotherapy Induced Androgen Receptor Splice Variant 7 in Patients with Castration-Resistant Prostate Cancer: A Tissue-based Analysis
Source: Sci Rep. 2019 Nov 14;9:16794. doi: 10.1038/s41598-019-53280-5 (PMC6856155; doi:10.1038/s41598-019-53280-5)

Taxane-based Chemotherapy Induced Androgen Receptor Splice Variant 7 in Patients with Castration-Resistant Prostate Cancer: A Tissue-based Analysis

Myungsun Shim[1], Yunlim Kim[2], Yangsoon Park[3], Hanjong Ahn[4,*]

[1] Department of Urology, Hallym University College of Medicine, Hallym University Sacred Heart Hospital, 22, Gwanpyeong-ro 170beon-gil, Dongan-gu, Anyang-si, Gyeonggi-do 14068, South Korea

[2]Institute for Innovative Cancer Research, Asan Institute for Life Science, Asan Medical Center, 88, Olympic-ro 43-gil, Songpa-gu, Seoul 05505, South Korea

[3] Department of Pathology, University of Ulsan College of Medicine, Asan Medical Center, 88, Olympic-ro 43-gil, Songpa-gu, Seoul 05505, South Korea

[4] Department of Urology, University of Ulsan College of Medicine, Asan Medical Center, 88, Olympic-ro 43-gil, Songpa-gu, Seoul 05505, South Korea

[*] Corresponding. hjahn@amc.seoul.kr

Supplementary information

Eq. A Exclusion criteria

Patients with following criteria were excluded from the analysis

1. Prostatic malignancies other than adenocarcinoma (e.g. prostate sarcoma)
2. History of previous radiation therapies
3. Missing data
4. Inappropriate specimen condition
5. Follow-up period of less than 1 year

Eq. B mRNA extractions and Real-time quantitative reverse transcription PCR

To minimize the heterogeneity of the tissue and to ensure obtaining pure cancer tissue, an experienced pathologist (Y Park) had confirmed the tissue and participated in tissue slicing. RNA extraction was performed from the routinely fixed TURP specimens using the RNase FFPE kit (Qiagen, Tübingen, Germany), from formalin-fixed paraffin embedded (FFPE) tissue sections (10 μm), which were directly cut from a FFPE tissue

sample block containing human prostate cancer (Pca). Total RNA (2 μg) was subjected to reverse transcription using the cDNA Synthesis Kit (Toyobo, Osaka, Japan) and the cDNA was subjected to real-time PCR with the ABI 7500 Fast sequence detector system (Applied Biosystems, Foster City, CA). The primers were designed as follows: ARfl forward, 5'-CAGTGGATGGGCTGAAAAT-3', reverse, 5'-AAGCGTCTTGAGCAGGATGT-3'; ARV7 forward, 5'-CCATCTTGTCGTCTTCGGAAATGTTATGAAGC-3', reverse, 5'-TTTGAATGAGGCAAGTCAGCCTTTCT-3'; GR forward, 5'-GCGATGGTCTCAGAAACCAAAC-3', reverse, 5'-GCAGAGGATAACTTCCTCTGTAATCTC-3'. The cycler was programmed with the following conditions (a) holding stage at 95°C for 20 seconds, followed by the (b) cycling stage with 40 cycles at 95°C for 3 seconds and subsequently at 60°C for 30 seconds. The subsequent melt curve stage comprises the following steps: 95°C for 15 seconds, a melt from 60°C for 1 minute to 95°C for 15 seconds with a ramp rate of 1%, and 60°C for 15 seconds. The PCR amplification efficiency and linearity for each gene, including the targeted and control genes, were tested. All the transcript levels of the receptors are shown as the copy number of the messenger RNA (mRNA), which was adjusted to the corresponding housekeeping gene (GAPDH) mRNA levels.

Eq. C Cell culture and Western blot analysis

C.1 Reagents and antibodies

Docetaxel was purchased from Sigma Aldrich. The following primary antibodies were used; AR, GR and GAPDH (Santa Cruz) and AR-V7 (Precision)

C.2 Cell culture and docetaxel treatment

The human prostate cancer cell line 22Rv1 was obtained from the American Type Culture Collection and maintained in RPMI 1640 (Invitrogen) with 5-10% heat-inactivated FBS, 100 units/ml of penicillin, and 100 μg/ml of streptomycin in a 5% CO_2 atmosphere at 37°C. Cells were seeded in 6-well plates ($6X10^5$ cells/well) containing 10% FBS media for 18-24h. Cells were incubated with 10nM docetaxel for 24 and 72 hours.

C.3 Western blot analysis

Cells lysates were prepared in lysis buffer containing protease inhibitor (Sigma Aldrich). The cell lysates were centrifuged at 13,000 x g for 10 min, and the supernatants were stored at 4°C. Then, the protein sample concentration was measured using Bradford protein assay (Bio-rad). Equal amounts of protein were subjected to sodium dodecyl sulphate (SDS)-polyacrylamide gel electrophoresis (PAGE), and transferred to PVDF membrane. The membranes were stained with Ponceau red to confirm equal sample loading and transfer. After blocking with 5% bovine serum albumin (BSA) for 1h at room temperature, the membranes were incubated with primary antibodies overnight at 4°C with shaking (100rpm). Then, membranes were incubated with peroxidase-conjugated secondary antibodies for 1h at RT. Immunoreactive bands were visualized on X-ray film using Immunobilion Western enhanced chemiluminescent (ECL) solution (Millipore Corp.). GAPDH were used as internal controls. Relative protein expression level was quantified using Image-J version 1.51 (National Institutes of Health) software and normalized to GAPDH (N=3).

Eq. D ‘High’ versus ‘Low’ transcription

For survival analysis, the patients were categorized as the “high transcription” group if the transcript level was equal to or greater than the median value of Group 1 or as the “low transcription” group if the transcript level was less than the median value of Group 1.

Eq. E The supplementary dataset is the original figure of western blot film for Figure 2.

Supplementary figure (A) shows protein expression changes of ARfl, ARV7 and GR after 24 and 72 hours after docetaxel treatment. (B) demonstrates the types of antibodies for western blot analysis. (C), and (D) is the original image before cropping gels/blots.

Eq. F Representative images of immunohistochemistry staining for a patient from each group. Staining intensity of ARV7 increased from Group 1 through to 3, while it decreased from Group 3 to 4. As for GR, staining intensity showed a steady increase from Group 1 through to 4. Tumor cells are depicted in the dashed line for Group 1. For other Groups, cancer cells are seen in the entire picture field (original magnification × 100).

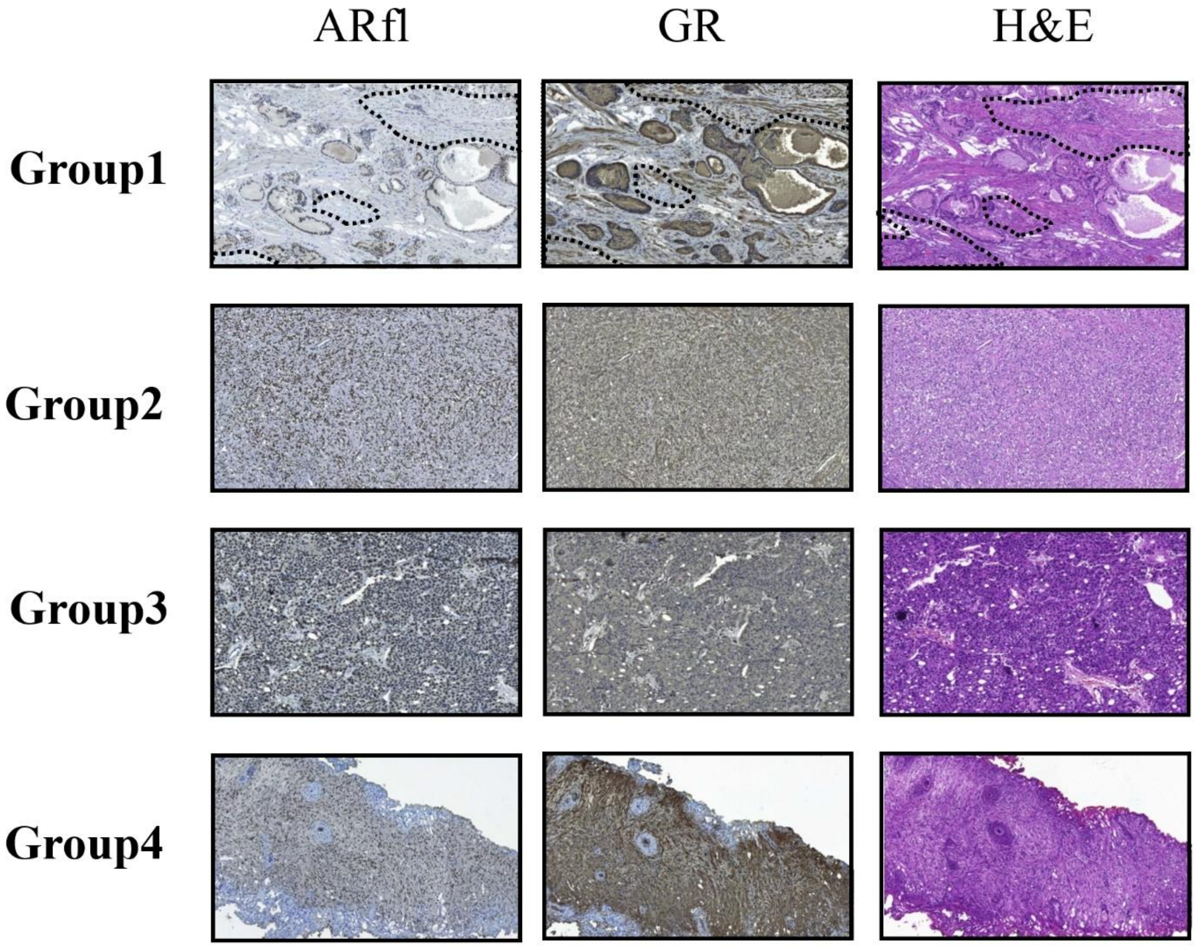

Eq. G Western blot analysis with ARV7 antibodies of individual patients from each group, β-actin was used for loading control. P1, P2, and P3 depicts ARV7 expression of patients from Group 1, 2, and 3, respectively. P4, P5, and P6 depicts those from Group 4 patients. Relatively strong expression of ARV7 is seen in Group 4 patients.

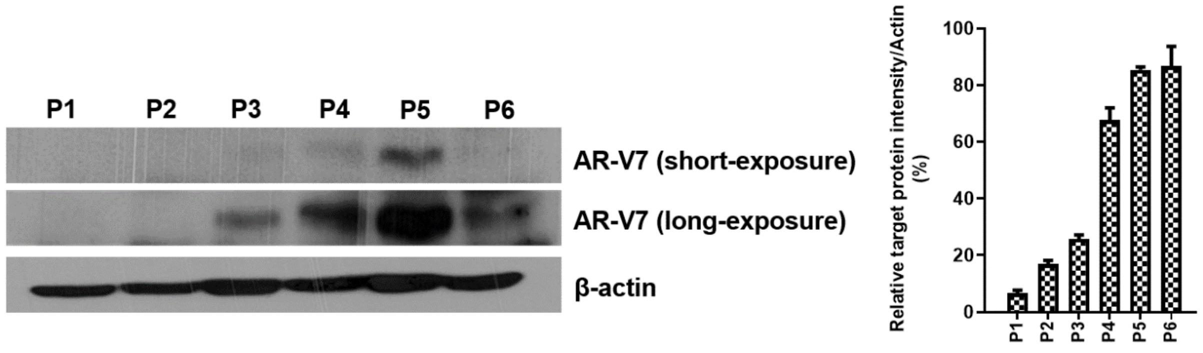

Supplement: Supplementary file 1 — Supplementary [file 41598_2019_53280_MOESM1_ESM.pdf]
